# Nematode neuropeptides as transgenic nematicides

**DOI:** 10.1371/journal.ppat.1006237

**Published:** 2017-02-27

**Authors:** Neil D. Warnock, Leonie Wilson, Cheryl Patten, Colin C. Fleming, Aaron G. Maule, Johnathan J. Dalzell

**Affiliations:** 1 Microbes & Pathogen Biology, The Institute for Global Food Security, School of Biological Sciences, Queen’s University Belfast, Belfast, United Kingdom; 2 Biology Department, University of New Brunswick, Saint John, NB, Canada; 3 Agri-Food and Biosciences Institute, Belfast, United Kingdom; The Ohio State University, UNITED STATES

## Abstract

Plant parasitic nematodes (PPNs) seriously threaten global food security. Conventionally an integrated approach to PPN management has relied heavily on carbamate, organophosphate and fumigant nematicides which are now being withdrawn over environmental health and safety concerns. This progressive withdrawal has left a significant shortcoming in our ability to manage these economically important parasites, and highlights the need for novel and robust control methods. Nematodes can assimilate exogenous peptides through retrograde transport along the chemosensory amphid neurons. Peptides can accumulate within cells of the central nerve ring and can elicit physiological effects when released to interact with receptors on adjoining cells. We have profiled bioactive neuropeptides from the neuropeptide-like protein (NLP) family of PPNs as novel nematicides, and have identified numerous discrete NLPs that negatively impact chemosensation, host invasion and stylet thrusting of the root knot nematode *Meloidogyne incognita* and the potato cyst nematode *Globodera pallida*. Transgenic secretion of these peptides from the rhizobacterium, *Bacillus subtilis*, and the terrestrial microalgae *Chlamydomonas reinhardtii* reduce tomato infection levels by up to 90% when compared with controls. These data pave the way for the exploitation of nematode neuropeptides as a novel class of plant protective nematicide, using novel non-food transgenic delivery systems which could be deployed on farmer-preferred cultivars.

## Author summary

Plant parasitic nematodes (PPN) reduce crop plant yield globally, undermining food security. Many of the chemicals used to kill these parasites are non-specific and highly toxic, and are being phased out of general use through governmental and EU regulation. The withdrawal of these chemicals is beneficial to the environment, but limits our ability to protect crops from infection. Efforts must now focus on developing environmentally safe PPN controls. PPNs can absorb various molecules directly from the environment into their nervous system, including peptides and proteins. Here we profiled the feasibility of using PPN neuropeptides, small signalling molecules, to interfere with normal PPN behaviour. We exposed PPNs to a variety of neuropeptides, and found that they could interfere with behaviours that are important to host-finding and invasion. We then developed soil-dwelling microbes that could generate and secrete these neuropeptides into the soil where the PPN infective juveniles are found. These transgenic microbes can protect host plants from infection, and represent a completely new approach to controlling PPNs in crop plants. Importantly, these neuropeptides appear to have no impact on other beneficial nematodes found in the soil.

## Introduction

Plant parasitic nematodes (PPNs) are responsible for an estimated 12.3% reduction in crop yield each year, which equates to losses of around $US80 billion worldwide [1, 2]. Traditionally PPNs have been controlled through the use of fumigant, carbamate and organophosphate nematicides which are being withdrawn over environmental health and safety concerns, through global and EU level directives [3]. The fumigant methyl bromide was used extensively to control PPN infestations for more than 60 years, however the identification of ozone-depleting characteristics was recognised within the Montreal Protocol which aimed to eliminate methyl bromide use by 2010 [4]. Likewise, dibromochloropropane (DBCP), a highly lipophilic brominated organochlorine was first used as a nematicide in the mid 1950’s before animal safety tests in the 1960’s demonstrated endocrine disrupting, and carcinogenic properties, alongside an increased incidence of developmental defects following exposure. Later studies further demonstrated strong mutagenic properties, and workers at the Occidental Chemical plant in California, which produced DBCP, displayed significantly higher rates of spermatogenic abnormalities relative to the rest of the population [5]. The carbamate nematicide aldicarb also triggers toxicity in non-target organisms through disruption of cholinergic neurons. Initial withdrawal of use across the USA in 1990 was followed by re-introductions to counteract a serious shortfall in alternative control options in 1995; similar dispensations have been afforded to EC states. The extensive withdrawal of frontline nematicides has left a significant shortfall in our ability to control PPNs.

Transgenic approaches could provide a cost-effective means of PPN control. Much effort has focused on the development of *in planta* RNA interference (RNAi) to silence PPN genes necessary for successful parasitism [6–9]. Whilst many such studies have shown promise, concerns surround the persistence of RNAi trigger-expressing traits. It remains to be established if DNA methylation and transcriptional silencing of double stranded (ds)RNA-expressing transgenes is an issue in plants other than *Arabidopsis thaliana* [10]. Efforts to inhibit PPN nutrient acquisition through transgenic expression of cystatins that inhibit intestinal protease activity have also proven an effective strategy [6]. The utility of peptide resistance traits has also been demonstrated [7], resulting in field level resistance and high target specificity [8]. Indeed, stacking peptide and cystatin resistance traits has proven extremely effective in plantain, triggering a 99% reduction in PPN infection levels at harvest, with a corresponding 86% increase in plantain yield [9].

Peptides have traditionally been viewed as poor drug candidates due to issues surrounding cellular uptake and half-life. However it has long been known that nematodes display an unusual neuronal uptake mechanism which is exploited by amphid dye-filling methods [11]. The amphid neurons assimilate exogenous peptides which subsequently accumulate in cells of the central nerve ring [11], where they can interact with available receptors.

Neuropeptides are highly enriched and conserved amongst nematodes, coordinating crucial aspects of physiology and behaviour [12–21]. The model nematode *Caenorhabditis elegans* encodes at least 113 neuropeptide genes, producing over 250 mature neuropeptides [16]. It is thought that this neurochemical diversity underpins the wide array of complex behaviours which are found within such neuroanatomically simple animals [16, 22]. Many neuropeptides are known to be expressed within the anterior neurons of nematodes [16, 22–24], and it is likely that their cognate receptors are expressed in these or adjacent cells. The retrograde transport of exogenous peptides suggest that these receptors could be amenable to activation through signalling molecules following their uptake from the external environment. Conceptually, the mining of native neuropeptide complements for novel nematicides is an attractive prospect, based on the *a priori* assumption of bioactivity. An additional positive quality of neuropeptides is their characteristically high potency when acting on cognate receptors [13, 25–30]. Furthermore, the high degree of phylogenetic sequence conservation suggests that neuropeptides could represent broad-spectrum nematicides as they share significant sequence similarity within and between parasite species [17, 22, 31, 32]. Disrupting PPN behaviour through the dysregulation of native neuropeptide signalling could hinder the development of resistance traits anchored on target receptor mutation. Selective pressure drives the propagation of drug target variants which escape agonism / antagonism, or the development of enhanced efflux mechanisms [33, 34]. Conceptually, the development of resistance to neuropeptides which coordinate crucial aspects of PPN biology would seem less likely.

Nematode neuropeptide complements are organised into three broad groupings: i) the FMRF-amide Like Peptides (FLPs); the INSulin like peptides (INSs); and iii) the Neuropeptide-Like Proteins (NLPs). FLPs represent the most widely studied and best understood family, characterised by a C-terminal RFamide motif, and are known to coordinate motor and sensory function [14, 16, 22]. In particular, C-terminal amidation is necessary for biological function, and so precludes FLPs from most transgenic delivery methods. INSs coordinate and integrate sensory signals with developmental circuits [35] and they share characteristic domain organisation and tertiary structure with vertebrate insulin peptides [16, 36–40]. Specific proteolytic processing requirements suggest that INSs do not represent ideal candidates for transgenic delivery methods. The NLPs represent the least studied grouping of neuropeptides, comprising every neuropeptide that does not conform to the biosynthetic and structural characteristics of FLPs or INSs and encompassing multiple peptide families. Little is known about their function in nematodes, however many NLPs are expressed in anterior neurons and do not appear to require post-translational modifications [20, 24, 40–45], making them more amenable to generation and delivery by transgenic systems than FLPs or INSs. A key gap in assessing the potential of unamidated NLPs as nematicides is the lack of data on their bioactivity in PPNs.

Here we aimed to characterise the NLP complements *in silico* for two economically important PPNs that display different modes of infection and parasitism, *M*. *incognita* and *G*. *pallida*. Subsequently we aimed to screen NLPs for their ability to dysregulate the normal behaviour of infective stage juveniles (J2s) when applied exogenously and, simultaneously, to develop and assess novel transgenic delivery methods as next generation plant protection platforms.

## Results

### BLASTp identification of predicted NLPs

Pro-peptide sequences of *C*. *elegans* NLPs predicted to be unamidated (no C-terminal glycine; uNLPs) were used as queries to conduct a BLASTp analysis of the predicted protein complements of both *M*. *incognita* and *G*. *pallida* [46, 47]. A total of four *nlp* genes encoding 25 predicted uNLPs were found within the *G*. *pallida* genome, and seven *nlp* genes encoding 28 predicted uNLPs within the *M*. *incognita* genome (Table 1).

**Table 1:** The predicted unamidated NLP complements of *Globodera pallida* and *Meloidogyne incognita*.

*Globodera pallida* NLPs*		*Meloidogyne incognita* NLPs*	
Gp-NLP-8a	FSDDELAAMPLNDLYLSSPYAFGPF	Mi-NLP-2	SSLASGRIGFRPA
Gp-NLP-8b	SFDRLEESAFFGQ	Mi-NLP-8a	AFDRLDVSPFDFDAMT
Gp-NLP-14a	ALDILESDDFGGF	Mi-NLP-8b	FNDDELSSLPFNFEYFPSLDTH
Gp-NLP-14b	ALDVMDGDGFGSFE	Mi-NLP-8c	AFDRLEDSGFFGL
Gp-NLP-14c	ALDTLEGDDFMGL	Mi-NLP-8d	AFDRLDNSFMLL
Gp-NLP-14d	LNELEGDGFMGLD	Mi-NLP-9a	AGARAFQRPDFDDASYEL
Gp-NLP-14e	ALDILDGDDFTGFS	Mi-NLP-9b	GGARTFLVGE
Gp-NLP-14f	ALDALEGNSFGF	Mi-NLP-9c	GGARAFAKLEE
Gp-NLP-15a	SFDSLTGPGFTGLDT	Mi-NLP-9d	GGARPFYEE
Gp-NLP-15b	SFDSFTGPGFTGLD	Mi-NLP-9e	GGARPFYGFFGGGEGTW
Gp-NLP-15c	SFDSFTGSGFTGLD	Mi-NLP-9f	GGGRYFIRPFADQ
Gp-NLP-15d	AAFDTDFTNYD	Mi-NLP-14a	ALDMLEGDDFIGMQ
Gp-NLP-15e	FEPFDGYGFNGFE	Mi-NLP-14b	ALDLMEGDGFGGGFD
Gp-NLP-15f	SFDSFMGPGFTGMD	Mi-NLP-14c	ALDMMEGDDFIGL
Gp-NLP-15g	AFDSFTGPGFTGMD	Mi-NLP-15a	AFDSFGTPGFTGFD
Gp-NLP-15h	AFDLFTGPGFTGMD	Mi-NLP-15b	SFDSFTGPGFTGLD
Gp-NLP-21a	GGARAFNFFAPPDE	Mi-NLP-15c	SFDSFVGKGFTGMD
Gp-NLP-21b	GGARAFNFFAPDE	Mi-NLP-15d	AFDSFGTPGFTGFD
Gp-NLP-21c	GGTRAFNFFVSDALPSSYE	Mi-NLP-15e	SAFDSFVGRGFTGMD
Gp-NLP-21d	SGIQTFRDDYDEKQAGEL	Mi-NLP-15f	AFDSFAGNGFTGFD
Gp-NLP-21e	AGGRLFRMVDLPDGDDFVPEG	Mi-NLP-15g	NFDAFMGPGFTGLD
Gp-NLP-21f	GGARPFYGGGYMDGTW	Mi-NLP-15h	AAFDSFVGRGFTGMD
Gp-NLP-21g	AGGRYFMRHFDDSPFAGWMA	Mi-NLP-18a	FAPRQFAFA
Gp-NLP-21h	GGARAFFGDADGPFNSASYWAP	Mi-NLP-18b	GMRNFAFA
Gp-NLP-21i	GGARAFNGAEETLLNVANLA	Mi-NLP-18c	SFGDYPFGSRTFAFA
		Mi-NLP-18d	AAENFDENNDIN
		Mi-NLP-18e	SSQFGGENSFARFAFA
		Mi-NLP-40	MVSWQPV

*, single letter annotation of amino acids.

### uNLPs dysregulate key behaviours of *M*. *incognita* J2s

**Fig 1:**
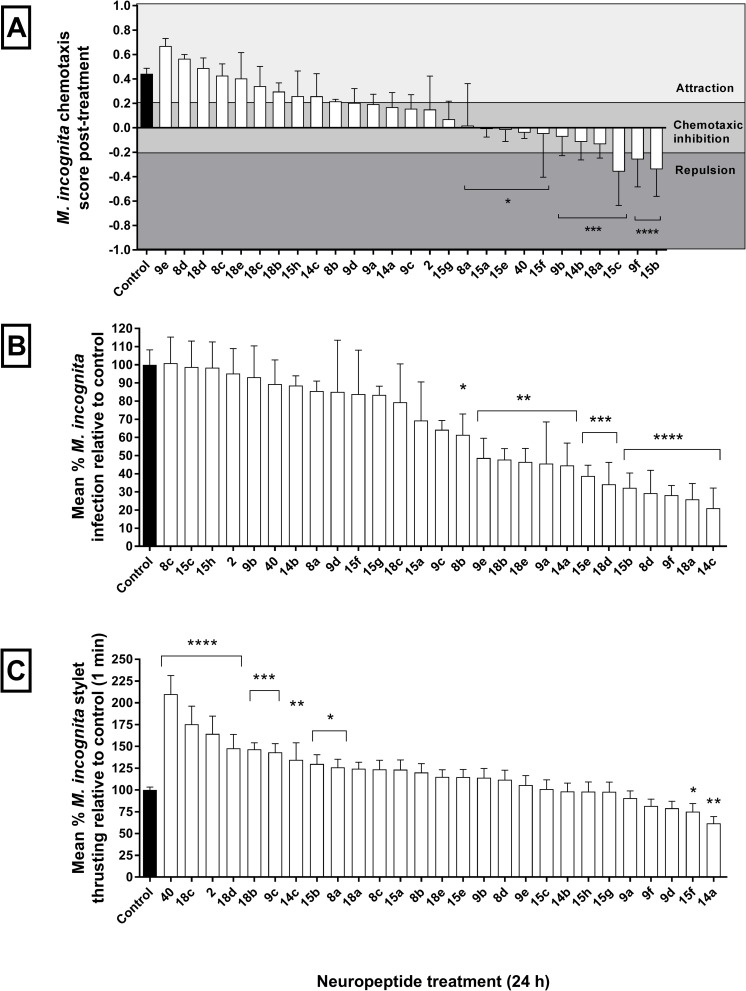
Exogenous neuropeptides disrupt normal *Meloidogyne incognita* chemotaxis, plant invasion and stylet thrusting. (A) 100 *M*. *incognita* infective stage juveniles (J2s) were incubated in selected uNLPs, and subsequently challenged with an agar plate chemosensory assay (plant root exudate attractant / water control). Each assay of 100 nematode juveniles was repeated ten times. (B) Ten tomato seedlings were individually challenged with 500 *M*. *incognita* J2s incubated in selected uNLPs. Numbers of invading *M*. *incognita* J2s were normalised against the negative control group, and expressed as a relative percentage. (C) 100 *M*. *incognita* J2s were incubated in selected uNLPs and the frequency of stylet thrusting in response to 5 mM serotonin was counted. Data were normalised to control treated groups. Data shown represent the mean±SEM. *, P<0.05; **, P<0.01; ***, P<0.001; ****, P<0.0001 (One-Way ANOVA & Fisher’s LSD; Graphpad Prism 6).

Predicted uNLPs were synthesised and screened against *M*. *incognita* and *G*. *pallida* J2s for plant protective qualities. Chemotaxis, host-invasion, and stylet thrusting behaviours were assayed following J2 exposure to 100 μM of each uNLP for 24 h. Eleven of 27 tested uNLPs were found to disrupt normal chemotaxis towards root exudate collected from tomato cv. Moneymaker (Fig 1A). Of particular interest is our observation that six of eight predicted *Mi-nlp-15* peptides inhibit chemosensation (Mi-NLP-15a/d, b, c, e, f). Analysis of the sequence similarity between these peptides suggest that the amino terminal variation of Mi-NLP-15g and h are responsible for observed functional differences. Two predicted *Mi-nlp-9* peptides also inhibit chemosensation (Mi-NLP-9b, f), however no clear amino acid differences correlate with functionality across predicted *nlp-9* peptides.

Likewise, 13 uNLPs were found to disrupt *M*. *incognita* host invasion (tomato cv. Moneymaker) compared to controls (Fig 1B). Multiple active uNLPs originated from single *nlp* genes, however no obvious amino acid conservation could predict bioactivity across *Mi-nlp-8*, *-9*, *-18* or *-15* peptides. In contrast, Mi-NLP14a and c share a common ALDMxEGDDFIGG motif. Three predicted uNLPs (Mi-NLP-15b, 9f, and 18a) inhibited both chemosensation and host invasion.

Eleven uNLPs were also found to disrupt the rate of serotonergic-induced *M*. *incognita* stylet thrusting (positively or negatively) compared with controls (Fig 1C). Multiple such uNLPs originated from *Mi-nlp-15* and *-18*, however no common amino acid motif could be found relative to the inactive predicted peptides from either gene. Mi-NLP-14a and c were observed to differentially stimulate the inhibition and excitation of stylet thrusting respectively. The amino acid sequences of both peptides suggest that this difference must be mediated by differences in the 5^th^ amino acid position, and/or differences at the carboxyl terminus. Mi-NLP-15b was the only predicted peptide to differentially regulate chemosensation, host invasion and stylet thrusting behaviours of *M*. *incognita* J2s (Refer to supplemental S1 Data).

### uNLPs dysregulate key behaviours of *G*. *pallida* J2s

**Fig 2:**
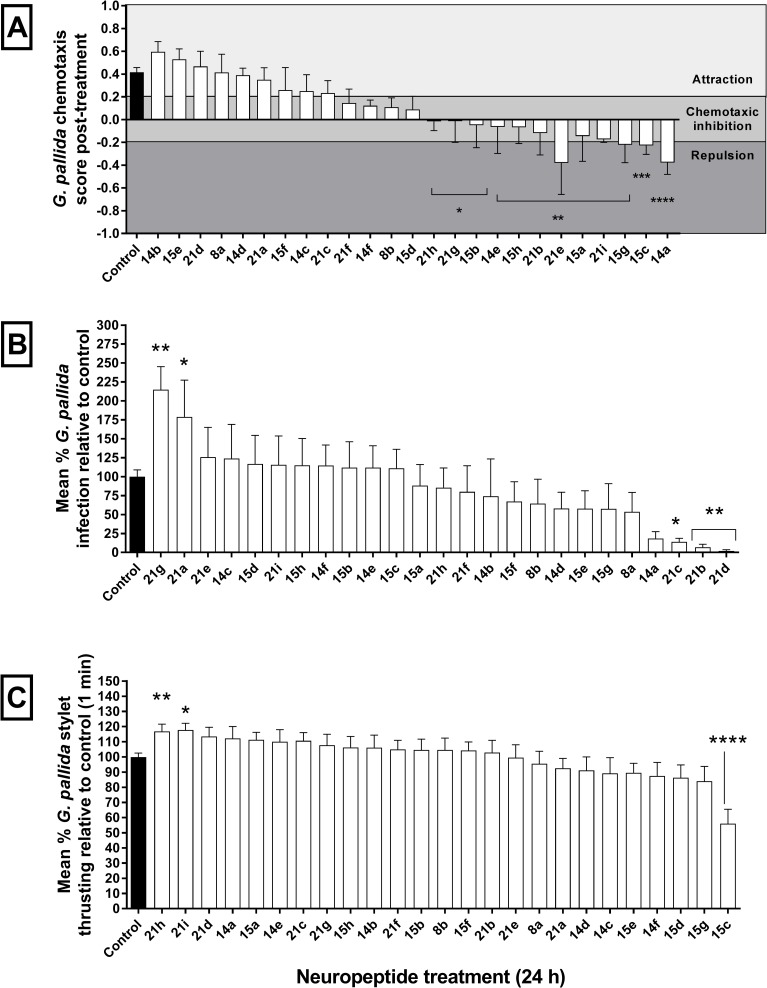
Exogenous neuropeptides disrupt normal *Globodera pallida* chemotaxis, plant invasion and stylet thrusting. (A) 100 *G*. *pallida* infective stage juveniles (J2s) were incubated in selected uNLPs, and subsequently challenged with an agar plate chemosensory assay (plant root exudate attractant / water control). Each assay of 100 nematode juveniles was repeated ten times. (B) Ten tomato seedlings were individually challenged with 500 *G*. *pallida* J2s incubated in selected uNLPs. Number of invading *G*. *pallida* J2s were normalised against the negative control group, and expressed as a relative percentage. (C) 100 *G*. *pallida* J2s were incubated in selected uNLPs and the frequency of stylet thrusting in response to 2 mM serotonin was counted. Data were normalised to control treated groups. Data shown represent the mean±SEM. *, P<0.05; **, P<0.01; ***, P<0.001; ****, P<0.0001 (One-Way ANOVA & Fisher’s LSD; Graphpad Prism 6).

12 of 25 tested uNLPs were found to disrupt chemotaxis of *G*. *pallida* J2s towards root exudate (tomato cv. Moneymaker), originating from *Gp-nlp14*, *-15* and *-21* (Fig 2A). Bioactivity of predicted peptides from *Gp-nlp-15* and *-21* does not correlate with an obvious amino acid sequence or motif, however Gp-NLP-14a and e peptides share an amino terminal ALDIL motif.

Five predicted *Gp-NLP-21* neuropeptides were found to disrupt *G*. *pallida* host invasion (tomato cv. Moneymaker) relative to controls (Fig 2B). No obvious amino acid sequence or motif was predictive for bioactivity relative to the other inactive *Gp-nlp-21* peptides. Both Gp-NLP-21b and g were found to inhibit both chemosensation and host invasion of *G*. *pallida* J2s.

Three uNLPs were also found to modulate serotonergic-induced stylet thrusting of *G*. *pallida* J2s relative to controls groups (Fig 2C). Gp-NLP-21h and -21i do not share any obvious amino acid similarity that is predictive for bioactivity relative to other inactive *Gp-nlp-21* peptides. Gp-NLP-21h, -21i and Gp-NLP-15c were found to inhibit chemosensation alongside modulating stylet thrusting rates (Refer to supplemental S1 Data).

### Mi-NLP-15b inhibits *M*. *incognita* chemotaxis and host invasion with high potency

**Fig 3:**
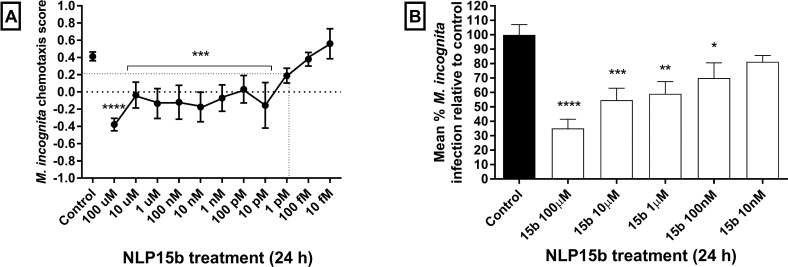
Mi- NLP-15b potently inhibits the chemotaxis and infectivity of *Meloidogyne incognita*. (A) Serial dilutions of Mi-NLP-15b indicate that J2 chemotaxis is inhibited by low picomolar concentrations. (B) Mi-NLP-15b significantly reduced J2 invasion levels at nanomolar concentrations. Data shown represent the mean±SEM. *, P<0.05; **, P<0.01; ***, P<0.001; ****, P<0.0001 (One-Way ANOVA & Fisher’s LSD; Graphpad Prism 6).

The potency of Mi-NLP-15b-induced disruption of chemotaxis and host invasion was assessed by exposing *M*. *incognita* J2s to various concentrations of synthetic Mi-NLP-15b for 24 h. Normal chemotaxis of *M*. *incognita* towards root exudate was inhibited across a range of dilutions, indicating high potency (Fig 3A). We found that *M*. *incongita* J2 invasion was also inhibited across a range of Mi-NLP-15b concentrations (Fig 3B; refer to supplemental S1 Data).

### Transgenic microbes secreting uNLPs protect plants from PPN invasion

**Fig 4:**
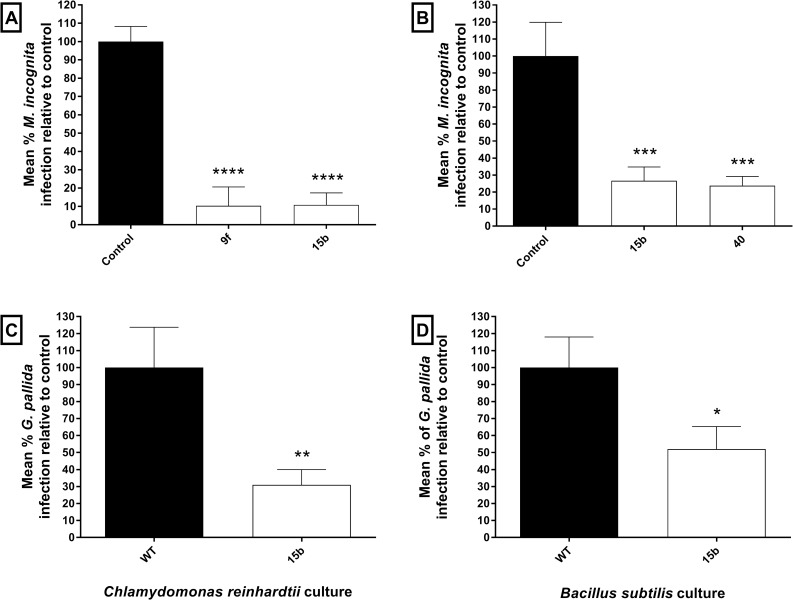
Transgenic microbes secreting uNLPs protect tomato against *Meloidogyne incognita* and *Globodera pallida*. (A) Nine independent *Chlamydomonas reinhardtii* transformants secreting two distinct nematode neuropeptides (Mi-NLP-9f and Mi-NLP-15b) significantly inhibited the ability of *M*. *incognita* J2s to infect tomato plants, with up to 90% protection. (B) *Bacillus subtilis* cultures secreting either Mi-NLP-15b or Mi-NLP-40 also conferred significant protection against *M*. *incognita* J2 invasion. (C) *C*. *reinhardtii* transformants secreting Gp-NLP-15b (identical to Mi-NLP-15b) significantly inhibited the ability of *G*. *pallida* J2s to invade tomato plants. (D) *B*. *subtilis* cultures secreting Gp-NLP-15b also protected tomato plants from *G*. *pallida* J2 invasion. Data shown represents mean±SEM. *, P<0.05; **, P<0.01; ***, P<0.001 (One-way ANOVA & Fisher’s LSD; Graphpad Prism 6).

Innoculation of *C*. *reinhardtii* cultures secreting selected uNLPs into the tomato invasion assay arena inhibited *M*. *incognita* invasion relative to untransformed *C*. *rehinhardtii*: Mi- NLP-9f (10.32% +/-10.32, p<0.0001), Mi-NLP-15b (10.82% +/-6.574, p<0.0001) (Fig 4A). Likewise, innoculation of *B*. *subtilis* cultures secreting selected uNLPs, significantly inhibited *M*. *incognita* invasion: Mi-NLP-15b (26.63% +/-8.12, p = 0.0003), Mi-NLP-40 (23.72% +/-5.448, p = 0.0002) (Fig 4B). *C*. *reinhardtii* expressing Gp-NLP-15b also inhibited *G*. *pallida* invasion relative to controls (30.95% +/-9.021, p = 0.0042) (Fig 4C). Similarly, innoculation with *B*. *subtilis* secreting Gp-NLP-15b inhibited *G*. *pallida* invasion relative to control groups (51.98% +/-13.29), p = 0.0203 (Fig 4D). Secretion of a His-tagged NLP-15b peptide from *B*. *subtilis* was confirmed by ELISA, indicating active secretion of 193.8 ±81.3 ng/ml in LB broth culture (refer to supplemental S2 Data).

### PPN uNLPs do not alter behaviours of non-target nematodes

BLAST was used to identify NLP-15b homologues across available expressed sequence tags (ESTs) or genomes of PPNs and non-target nematode species. PPNs with diverse life history traits share high levels of NLP-15b sequence similarity, however sequence similarity is reduced in non-target nematode species (Table 2).

**Table 2:** Sequence alignment of NLP-15b in selected parasitic nematode species and the free living nematode *C*. *elegans*.

Nematode Species	NLP-15b sequence*
*Meloidogyne incognita*	SFDSFTGPGFTGLD
*Meloidogyne javanica*	SFDSFTGPGFTGLD
*Meloidogyne hapla*	SFDSFTGPGFTGLD
*Meloidogyne chitwoodi*	SFDSF**M**GPGFTGLD
*Globodera pallida*	SFDSFTGPGFTGLD
*Globodera rostochiensis*	SFDSFTGPGFTGLD
*Heterodera glycines*	SFDSFTGPGFTGLD
*Pratylenchus penetrans*	SFDSF**M**GPGFTGLD
*Radopholus similis*	SFDSF**M**GPG**L**TGLD
*Steinernema carpocapsae*	**A**FDSF**M**G**S**GFTG**M**D
*Pristionchus pacifies*	SFD**T**FGG**VR**F**SP**L**E**
*Caenorhabditis elegans*	**A**FDS**LA**G**S**GF**GAFN**

*single letter annotation of amino acids. **Underlined** amino acids denote a deviation from the consensus sequence.

**Fig 5:**
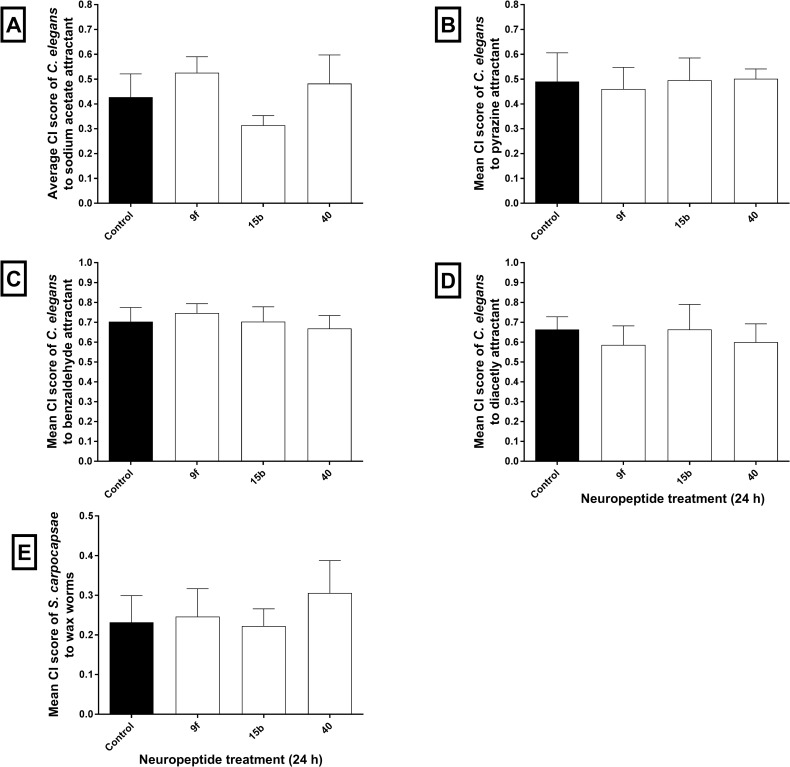
Plant parasitic nematode (PPN) unamidated neuropeptide-like proteins (uNLPs) do not alter *Caenorhabditis elegans* chemotaxis or *Steinernema carpocapsae* host-finding Chemotaxis of mixed stage *C*. *elegans* towards the attractants sodium acetate (A), pyrazine (B), benzaldehyde (C), and diacetyl (D) are unaffected by exposure to selected PPN uNLPs. (E) Chemotaxis of *S*. *carpocapsae* towards the insect host *Galleria mellonella* is also unaffected by exposure to selected PPN uNLPs. Data shown represent mean ±SEM (One-way ANOVA & Fisher’s LSD; Graphpad Prism 6).

Incubation of mixed-stage *C*. *elegans* in selected PPN uNLPs (100 μM, 24 h) had no statistically significant impact on chemotaxis towards the attractants: sodium acetate, pyrazine, benzaldehyde or diacetyl, relative to controls (Fig 5D). Exposure of *S*. *carpocapsae* infective juveniles (IJs) to selected PPN uNLPs also had no statistically significant impact on insect host-finding (Fig 5E).

## Discussion

We have identified seven *nlp* genes that putatively encode 27 mature unamidated peptides in the root knot nematode, *M*. *incognita* (*Mi-nlp-2*, *-8*, *-9*, *-14*, *-15*, *-18*, *-40*). Likewise, four *nlp* genes predicted to encode 24 mature unamidated peptides were identified in the potato cyst nematode, *G*. *pallida* (*Gp-nlp-8*, *-14*, *-15*, *-21*) (Table 1). Several predicted unamidated NLPs share high levels of amino acid sequence similarity between *M*. *incognita* and *G*. *pallida*, with one predicted peptide, designated NLP-15b, perfectly conserved between the two. Indeed, NLP-15b is highly conserved at the sequence level across PPN species with diverse life history traits; less sequence similarity is observed between NLP-15b from PPNs and non-target species such as *S*. *carpocapsae*, *C*. *elegans* or *P*. *pacificus* for example (see Table 2).

Selected *M*. *incognita* and *G*. *pallida* peptides had a negative impact on PPN chemosensation and host-finding behaviours, but not on chemosensory or host-finding behaviours of mixed stage *C*. *elegans* or *S*. *carpocapsae* infective juveniles (Figs 1, 2 and 5). This may be due to NLP sequence dissimilarity, or to different peptide uptake efficiencies between species. The attractants used to assay *C*. *elegans* chemotaxis operate via distinct neuroanatomical and biochemical pathways; sodium acetate is detected by the ASE neurons, benzaldehyde by the AWC neurons and prazine and diacetyl are both detected by the AWA neuron. The ASE, AWC and AWA neurons mediate aspects of water soluble and volatile chemotaxis in *C*. *elegans* [48, 49]. Off-target NLP impacts were also assessed as a factor of host-finding ability in *S*. *carpocapsae* which will involve numerous neuroanatomical and biochemical pathways. Whilst these data on *C*. *elegans* and *S*. *carpocapsae* are far from exhaustive, they suggest that neuropeptide treatments that produce strong disruptive effects on the behaviours of *M*. *incognita* and *G*. *pallida* may be specific to PPNs.

PPNs use a hollow protrusible stylet in order to pierce plant cells on entry to the plant root, and to secrete various parasitism effectors related initially to cell wall degradation, and subsequently to the re-programming of plant cells into giant cell (RKN) or syncytial (PCN) feeding sites. Our data reveal that both agonistic and antagonistic disruption of stylet thrusting can reduce host invasion rates, however modulation of stylet thrusting does not always correlate with modified invasion behaviour under the conditions tested here. Mi-NLP-14c, Mi-NLP-18b, and -18d agonise serotonergic stylet thrusting, have no negative impact on J2 chemosensory ability, and yet also reduce host invasion rates. Mi-NLP-15f reduces stylet thrusting rate, but does not impact on host invasion, whereas Mi-NLP-14a reduces stylet thrusting rate and does inhibit host invasion. None of the three uNLPs that dysregulate stylet thrusting in *G*. *pallida* have an impact on host invasion rate. We hypothesise that enhanced stylet thrusting rate may be beneficial for initial invasion events, however it seems likely that coordinated stylet thrusting behaviour is more beneficial during feeding site development for example. Our data do not point to an obvious outcome in this regard, however we do find that dysregulation of behaviour tends to lower plant invasion levels of both *M*. *incognita* and *G*. *pallida* J2s.

Whilst it is tempting to extrapolate something on native NLP functionality from these data, we do not know if the aberrant phenotypes observed are due to interactions between tested NLPs and their cognate receptors. However, we do observe that exogenous NLPs can interact with endogenous neurophysiological circuits, interfering with host-finding, invasion and serotonergic stylet-thrusting behaviours of both *M*. *incognita* and *G*. *pallida* juveniles (Figs 1 and 2). This supports our initial hypothesis that nematode neuropeptides represent a valuable repository of nematicide candidates, which may elicit broad-spectrum activities against PPN species, but not off-target nematode species. Serial dilution of Mi-NLP-15b inhibited *M*. *incognita* chemosensation at concentrations as low as 10 pM, demonstrating high uNLP potency, which is a known characteristic of interactions between nematode neuropeptides and their cognate receptors [13, 25–30, 50, 51] (Fig 3). While the potency of this peptide would support the specificty of the associated phenotypic impact, we advise some caution when interpreting these data as indicative of NLP function within either *M*. *incognita* or *G*. *pallida* J2s due to the potential for peptide interaction with other, non-cognate receptors.

In order to further assess the efficacy of exogenous NLPs as nematicides, we developed two transgenic synthesis and delivery systems which could be deployed in the field, potentially through seed treatments or soil amendments. Gram positive *Bacillus* spp. are a major component of rhizosphere microbial communities [52, 53], and are frequently categorised as Plant Growth Promoting Rhizobacteria (PRPR) [54, 55]; *B*. *subtilis* has also been shown effective in controlling *Meloidogyne* species [56]. More generally, *B*. *subtilis* represents an important organism for many biotechnology applications, and is classified as GRAS (generally regarded as safe) by the FDA [57, 58]. It is increasingly well served by the development of synthetic biology tools [59], and can persist in soil for long periods through the production of spores [60]. We modified *B*. *subtilis* to secrete a number of PPN NLPs, and found that transformed *B*. *subtilis* cultures confer significant levels of protection on tomato cv. Moneymaker against both *M*. *incognita* and *G*. *pallida* infective juveniles (Fig 4). This proof of concept demonstration employed a commercial *B*. *subtilis* strain and signal peptide sequence. It has however been reported that signal peptide identity can have a significant influence on the level of protein / peptide secreted by *B*. *subtilis* [61, 62]. Unfortunately, we were unable to raise a suitable antisera to NLP-15b over several commercial synthesis rounds, due to the lack of NLP-15b immunogenicity. This restricted our ability to confirm amphidial uptake of the uNLPs, and to quantify microbial secretion of the uNLPs. Whilst we aimed to deliver proof of principle for this approach using commercially available and independently validated microbial synthesis and secretion systems, we confirmed secretion of a His-tagged NLP-15b from *B*. *subtilis* by ELISA (S2 Data). We anticipate that signal peptide optimisation efforts could increase secretion and correspondingly enhance plant protection levels. Likewise, assessing other rhizobacteria strains may enhance efficacy. The secretion of uNLP nematicides could also be more targeted if driven by a plant root exudate-responsive promoter [63, 64, 65, 66].We also utilised the soil-dwelling microalgae, *C*. *reinhardtii* as a novel synthesis and delivery platform. Like *B*. *subtilis*, *C*. *reinhardtii* benefits from an improving suite of synthetic biology tools [67]. *C*. *reinhardtii* cultures secreting selected PPN NLPs also provided significant levels of protection to tomato cv. Moneymaker when challenged by either *M*. *incognita* or *G*. *pallida* infective juveniles (Fig 4).

The NLP screening approach employed here may underestimate the efficacy achievable through a continuous transgenic delivery (Figs 1 and 2). For example, exogenous NLP-15b exposure inhibits *G*. *pallida* chemotaxis, but does not inhibit host invasion (Fig 2). However, when NLP-15b is delivered continuously to *G*. *pallida* infective juveniles via microbial secretion, we observe a significant inhibition of tomato invasion relative to J2s exposed to unmodified *B*. *subtilis* (Fig 4). This discrepency may be due to the recovery of *G*. *pallida* infective juveniles over the 24 hour timecourse of the tomato invasion assay. We expect that this may result in some false negative determinations in our NLP pre-screening approach.

Our data demonstrate that unamidated NLPs represent a new class of potent and specific plant protective nematicide that could be deployed as a transgenic trait in crop plants, or through soil microorganisms such as the *B*. *subtilis* and *C*. *reinhardtii* systems developed here. In particular, these non-crop delivery approaches could facilitate rapid deployment to many different crop plant species and cultivars. A key consideration in the development of PPN resistance traits must be the maintenance of genetic diversity across crop cultivars and isolates. This reduces the chance of widespread pathology from other pests as a result of genetic bottlenecks introduced by a single preferred transgenic cultivar.

## Materials and methods

### BLAST identification of PPN uNLPs

The predicted NLP complement of *C*. *elegans* [16] was used in a simple BLASTp and tBLASTn analysis of available genomic / transcriptomic sequence data of *G*. *pallida* and *M*. *incognita* [46, 47]. All returned hits were curated by eye, and NLPs identified as per McVeigh *et al*. [17].

### PPN maintenance

*M*. *incognita* were maintained in tomato plants (cv. Moneymaker) under greenhouse conditions. 8 weeks post infection *M*. *incognita* eggs were harvested from the roots by washing away excess soil and by briefly treating cleaned roots in 5% sodium hypochlorite to soften the root tissue and release the eggs. Eggs were cleaned from debris by passage through nested sieves (180 micron, 150 micron and 38 micron) and washed thoroughly with water. Eggs were separated from remaining soil / silt by centrifugation (2000 rcf for 2 minutes) in 100% sucrose solution and collected in a thin layer of spring water (autoclaved and adjusted to pH 7). Eggs were treated in antibiotic / antimycotic solution (Sigma) overnight, placed in a nylon net with a 38 micron pore size, immersed in spring water and maintained in darkness at 23°C, until infective juveniles emerged. Freshly hatched juveniles were used for each assay.

*G*. *pallida* were maintained in potato (cv. Cara) at the Agri-Food and Biosciences Institute (AFBI), Belfast. Soil was collected surrounding potato roots, dried for one week and washed through sieves to collect cysts. Cysts were incubated in potato root diffusate in the dark at 17°C until infective juveniles emerged. Freshly hatched juveniles were used for each assay.

### PPN uNLP screen

Predicted uNLPs from both *M*. *incognita* and *G*. *pallida* were synthesised by EZBiolab and dissolved into pH adjusted ddH_2_O to make a 5 mM stock which was aliquoted and stored at -20°C. J2s of both *M*. *incognita* and *G*. *pallida* were incubated for 24 hours in 200 μl of each peptide in a 24 well plate (SPL Lifesciences, South Korea) at a defined concentration.

### PPN uNLP screen: Chemosensory assays

A 60 mm Petri dish was divided into two segments, a positive and a negative side, with a 0.5 cm 'dead zone' either side of the centre point. The petri dish was filled with 15 ml of 0.25% w/v agar which was allowed to solidify. 3 ml of 0.25% w/v agar slurry in spring water (pH 7, agitated with a magnetic stirrer for several hours to give a smooth consistency) was added to the petri dish and spread evenly over the surface. Root diffusate (attractant) and water only (control) 0.25% agar plugs were embedded in the agar slurry, either side of the assay arena. Root diffusate was collected from 10 tomato plants, aged 3–6 weeks in 1 litre pots, by pouring 500 ml of ddH_2_0 through the soil three times. Diffusate from each plant was combined, filter sterilised and stored at 4°C for a maximum of 1 month. Root diffusate agar plugs were made by melting 1.25% agar in ddH_2_0, cooling to 50°C before mixing with 4 parts of root diffusate. The agar was then allowed to solidify at room temperature. 100 uNLP pre-treated *M*. *incognita* or *G*. *pallida* J2s were added by pipette to the centre of the plate. J2s which moved out of the 'dead zone' after 3 hours were counted and their location (+/-) scored. The distribution of J2s were used to create a chemotaxis index [68] for each plate, which formed one replicate, a total of 10 replicates where completed for each uNLP treatment.

### PPN uNLP screen: Tomato invasion assays

Tomato seeds were sterilised with 2.5% NaOCl for 15 minutes, washed 5 times in ddH_2_0 and germinated on 0.5% Murashige and Skoog plates at 23°C. An agar slurry was prepared by autoclaving 0.55% (w/v) agar (using autoclaved spring water adjusted to pH 7) which was mechanically agitated overnight until it had a smooth consistency. Invasion assays were performed by mixing 500 pre-treated *M*. *incognita* or *G*. *pallida* J2s with agar slurry and a single tomato seedling (2 days post germination) in a 6 well plate. Assays were left at 23°C for 24 hours in the case of *M*. *incognita* and at 18°C for 24 hours in the case of *G*. *pallida* under a 16 hour light and 8 hour darkness cycle. Seedlings were stained using acid fuschin [69] and the number of nematodes within the roots counted. At least five seedlings were used for *M*. *incognita* infections assays, with at least 15 seedlings used for *G*. *pallida* infections assays (due to increased variation).

### PPN uNLP screen: Stylet thrusting assays

Stylet thrusting assays where performed by incubating 100 *M*. *incognita* or *G*. *pallida* J2s for 15 minute in 5 mM or 2 mM serotonin (Sigma Aldrich, USA), respectively. J2s were placed on a glass slide and stylet thrusts were counted for randomly selected J2s, for 1 minute each. Counts for a given cohort of J2s were taken in a maximum interval of 15 minutes. Longer counting intervals, making for longer serotonin incubations, yielded inconsistent results. At least 30 J2s were counted for each neuropeptide treatment.

### *B*. *subtilis* and *C*. *reinhardtii* plant protection assays

*B*. *subtilis* were grown overnight in LB media containing ampicillin (100 μg/ml) at 37°C with shaking, and harvested in the log phase of growth determined by measuring OD_600nm_. Five ml of culture at 0.5 OD was spun down and the pellet mixed with 3 ml of agar slurry and 500 J2s from either *G*. *pallida* or *M*. *incognita*. *C*. *reinhardtii* clones were grown at 23°C with shaking, cultures in the log phase were measured at OD_750_ and 5 ml of culture at 0.5 OD was pelleted by centrifugation. *C*. *reinhardtii* pellets were mixed with 3 ml of agar slurry and 500 J2s from either *G*. *pallida* or *M*. *incognita*. Plant invasion assays were performed as described above.

### *C*. *elegans* culture and assays

*C*. *elegans* wild-type N2 Bristol strain were obtained from the *C*. *elegans* Genomics Center and maintained on a *Escherichia coli* (strain OP50) lawn on nematode growth medium (NGM) agar plates (3 g/l NaCl, 17 g/l agar, 2.5 g/l peptone, 5 mg/l cholesterol, 25 mM KH_2_PO_4_ (pH 6.0), 1 mM CaCl_2_, 1 mM MgSO_4_) at 20°C [70]. Chemotaxis assays were performed in a 9 cm diameter Petri dish on NGM agar which was split into a positive and negative side with a central ‘dead zone’ of 1.5 cm diameter. 100 mixed-staged *C*. *elegans* were washed three times in M9 buffer and soaked in 100 μM PPN uNLP, or M9 vehicle control for 24 hours. 2 μl of 50 mM sodium acetate, 0.5% pyrazine, 0.5% benzaldehyde or 0.5% diacetyl was spotted onto the positive side, 2 μl of ddH_2_0 was spotted onto the negative side. Pyrazine, benzaldehyde and diacetyl volatile attractants were assayed immediately whereas the water soluble sodium acetate was assayed 18 hours following addition to the plate. Assays were maintained in the dark at 20°C, and counted after 1 hour. Eight replicates were conducted for each *C*. *elegans* attraction assay.

### *S*. *carpocapsae* culture and host-finding assay

*S*. *carpocapsae* were cultured in *Galleria mellonella* at 23°C. Infective juveniles (IJs) were collected using a White trap [71] in PBS. Freshly emerged IJs were used for each assay. 100 IJs were incubated for 24 hours in 100 μM of selected uNLPs, and host-finding assays (n = 5) performed as in Morris et al. [45].

### Construction of uNLP expression/secretion plasmids

Codon optimised DNA sequences coding for the desired neuropeptide flanked by restriction sites necessary to clone into the *C*. *reinhardtii* expression vector pChlamy_3 (Life Technologies, USA) or the *B*. *subtilis* expression vector pBE-S (Clontech, USA) were synthesised by GeneArt Gene Synthesis (Life Technologies, USA).

### Transformation of *C*. *reinhardtii*

uNLP secretion inserts, and vector pChlamy_3 were digested using KpnI/XbaI (New England Biolabs, USA), ligated using T4 ligase (New England Biolabs, USA), and cloned into *Escherichia coli* One Shot TOP10 chemically competent cells (Life Technologies, USA) following manufacturer’s instructions. Ampicillin (Sigma Aldrich, USA) was used to select *E*. *coli* containing the pChlamy_3 plasmid, which was subsequently extracted using the High Pure Plasmid Isolation Kit (Roche) and sequenced (Eurofins Genomics, UK) to identify correct clones. *C*. *reinhardtii* was transformed by electroporation following manufacturer’s instructions (GeneArt *Chlamydomonas* Engineering Kit, Life Technologies) and individual colonies grown on TAP-Agar-Hygromycin plates (10 μg/mL) (Sigma Aldrich, USA) at 23°C. Colonies were picked and grown at 23°C in 100 ml TAP growth media (Invitrogen, USA) with constant orbital agitation. qRT-PCR was performed to identify clones with the highest level of uNLP expression, which were then selected for downstream assays (pChlamy universal FWD: CACTTTCAGCGACAAACGAG, *nlp-15b* REV: CTACTAGTCGAGGCCGGTA; Mi-*nlp-9f* REV: GAACGGGCGGATGAAGTAG).

### Transformation of *B*. *subtilis*

uNLP secretion inserts, and vector pBE-S were digested using XbaI/MluI (New England Biolabs, USA), ligated using T4 ligase (New England Biolabs, USA), and cloned into *E*. *coli* One Shot TOP10 chemically competent cells (Life Technologies, USA) following manufacturer’s instructions. Ampicillin (Sigma Aldrich, USA) was used to select *E*. *coli* containing the pBE-S plasmids, which were subsequently extracted using the High Pure Plasmid Isolation Kit (Roche) and sequenced (Eurofins Genomics, UK) to identify correct clones. *B*. *subtilis* RIK1285 competent cells (Takara, USA) were transformed according to manufacturer’s instructions and grown overnight at 37°C on kanamycin selective plates (10 μg/mL) (Sigma Aldrich, USA). Individual colonies were picked and grown in LB broth overnight at 37°C. qRT-PCR (pBE-S universal FWD: GGATCAGCTTGTTGTTTGCGT, *nlp-15b* REV: CCTGGCCCAGTGAAAGAGTC, *Mi-nlp-40* REV: TACCGGCTGCCAAGATACCA) was performed to confirm the expression of uNLP secretion cassettes.

### Confirmation of NLP-15b secretion from *B*. *subtilis*

Codon optimised NLP-15b, tagged with six histidine residues and an upstream aprE signal peptide, were cloned into the pBE-S vector (GeneArt, Life Technologies) and transformed into *B*. *subtilis* following manufacturer’s instructions (Takara Bio, Inc.). NLP-15b transformed B. subtilis were grown at 37°C in 50 ml of LB (kanamycin 10 μg/ml) and wild type *B*. *subtilis* in 50 ml of LB without selection. Once growth passed the exponential phase (OD 660) one tablet of cOmplete Protease Inhibitor Cocktail (Roche) was added to 10 ml of bacterial suspension and allowed to dissolve. Bacteria were removed from the LB by centrifugation at 10,000g for 10 minutes. Supernatant was collected and peptides were isolated by a MWCO 3 kDa filter (Amicon, Sigma). Histidine tagged peptide concentration assessed using the His Tag protein ELISA Kit (Cell Biolabs, Inc.) following manufacturer’s instructions. The ELISA results were measured (OD 450) using the FLUOstar Omega microplate reader (BMG Labtech). A line of best fit was plotted and the slope used to calculate the concentration of peptide across individual samples (n = >11).

### Statistical analysis

Data pertaining to behavioural and invasion assays were assessed by Brown-Forsythe and Bartlett’s tests to examine homogeneity of variance between groups. One-way ANOVA was followed by Fisher’s Least Significant Difference (LSD) test. All statistical tests were performed using GraphPad Prism 6.

## Supporting information

S1 Data Summary data for PPN behavioural assays. (XLSX)

S2 Data Summary data for HIS-tagged NLP-15b secretion from *B*. *subtilis*. (DOCX)
