# Supplementary figures and images for: Nematode neuropeptides as transgenic nematicides
Source: PLoS Pathog. 2017 Feb 27;13(2):e1006237. doi: 10.1371/journal.ppat.1006237 (PMC5344539; doi:10.1371/journal.ppat.1006237)

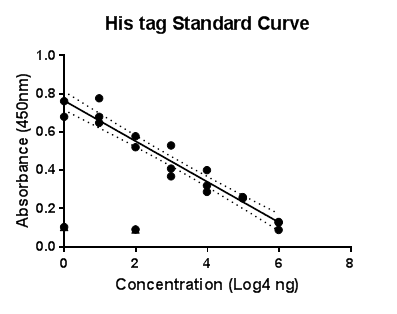


R square : 0.9458

YIntercept : 0.7648

Slope : -0.1062


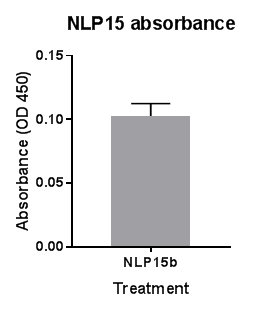


Predicted concentration: 193.8 ng/ml +/-81.3

Supplement: S2 Data — (DOCX) [file ppat.1006237.s002.docx]
